# Supplementary material for: Accuracy of the Infectious Diseases Society of America and British Thoracic Society Criteria for Acute Pneumonia in Differentiating Chemical and Bacterial Complications of Aspiration in Comatose Ventilated Patients Following Drug Poisoning
Source: Antibiotics (Basel). 2024 May 27;13(6):495. doi: 10.3390/antibiotics13060495 (PMC11200670; doi:10.3390/antibiotics13060495)
Supplement: Supplementary file 1 [file antibiotics-13-00495-s001.zip › antibiotics-3012505-supplementary/Table S1.pdf]

Table S1. Bacteria isolated from positive culture of tracheal samples.

Haemophilus influenzae, n (%)	10 (29)
Streptococcus pneumoniae, n (%)	8 (24)
Methicillin susceptible Staphylococcus aureus, n (%)	11 (32)
Klebsiella pneumoniae, n (%)	3 (9)
Klebsiella oxytoca, n (%)	3 (9)
Enterobacter aerogenes, n (%)	1 (3)
Pseudomonas aeruginosa, n (%)	2 (6)
Hafnia alvei, n (%)	1 (3)
Escherichia coli, n (%)	4 (12)
Proteus mirabilis, n (%)	1 (3)
Streptococcus agalactiae, n (%)	1 (3)
Citrobacter freundii, n (%)	1 (3)
