# Supplementary figures and images for: Accuracy of the Infectious Diseases Society of America and British Thoracic Society Criteria for Acute Pneumonia in Differentiating Chemical and Bacterial Complications of Aspiration in Comatose Ventilated Patients Following Drug Poisoning
Source: Antibiotics (Basel). 2024 May 27;13(6):495. doi: 10.3390/antibiotics13060495 (PMC11200670; doi:10.3390/antibiotics13060495)

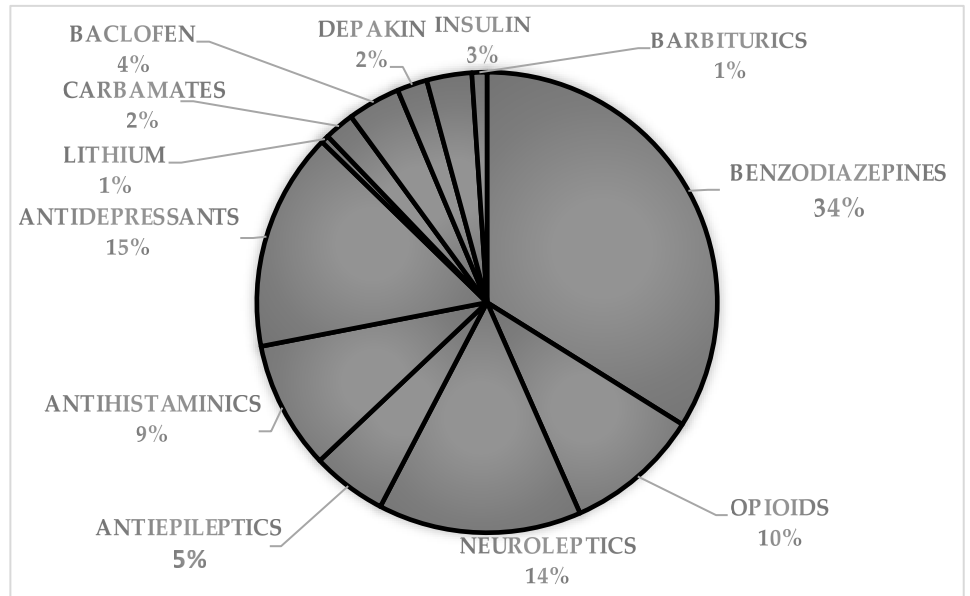

**Figure 1.** Repartition of drugs involved in poisoning

Supplement: Supplementary file 1 [file antibiotics-13-00495-s001.zip › antibiotics-3012505-supplementary/Figure S1.pdf]
